# The Texas flood registry: a flexible tool for environmental and public health practitioners and researchers

**DOI:** 10.1038/s41370-021-00347-z

**Published:** 2021-06-26

**Authors:** Marie Lynn Miranda, Rashida Callender, Joally M. Canales, Elena Craft, Katherine B. Ensor, Max Grossman, Loren Hopkins, Jocelyn Johnston, Umair Shah, Joshua Tootoo

**Affiliations:** 1 Children’s Environmental Health Initiative, University of Notre Dame, South Bend, IN USA; 2 Department of Applied and Computational Mathematics and Statistics, University of Notre Dame, South Bend, IN USA; 3 Children’s Environmental Health Initiative, Rice University, Houston, TX USA; 4 Environmental Defense Fund, Austin, TX USA; 5 Department of Statistics, Rice University, Houston, TX USA; 6 Houston Health Department, Houston, TX USA; 7 Harris County Public Health, Houston, TX USA; 8 Washington State Department of Health, Olympia, WA USA

**Keywords:** Air pollution, Climate change, Geospatial Analyses

## Abstract

**Background:**

Making landfall in Rockport, Texas in August 2017, Hurricane Harvey resulted in unprecedented flooding, displacing tens of thousands of people, and creating environmental hazards and exposures for many more.

**Objective:**

We describe a collaborative project to establish the Texas Flood Registry to track the health and housing impacts of major flooding events.

**Methods:**

Those who enroll in the registry answer retrospective questions regarding the impact of storms on their health and housing status. We recruit both those who did and did not flood during storm events to enable key comparisons. We leverage partnerships with multiple local health departments, community groups, and media outlets to recruit broadly. We performed a preliminary analysis using multivariable logistic regression and a binomial Bayesian conditional autoregressive (CAR) spatial model.

**Results:**

We find that those whose homes flooded, or who came into direct skin contact with flood water, are more likely to experience a series of self-reported health effects. Median household income is inversely related to adverse health effects, and spatial analysis provides important insights within the modeling approach.

**Significance:**

Global climate change is likely to increase the number and intensity of rainfall events, resulting in additional health burdens. Population-level data on the health and housing impacts of major flooding events is imperative in preparing for our planet’s future.

## Introduction

Hurricane Harvey made landfall on August 26, 2017 in Rockport, Texas, just southwest of Houston. Hurricane Harvey conjures vivid images of huge swaths of flooded areas, with dramatic boat and helicopter rescues of people of all ages and backgrounds. Dumping over 50 inches of rain on the Houston region, Harvey was the most significant tropical cyclone rainfall event in United States history, resulting in unprecedented flooding, including 300,000 confirmed flooded structures [1]. Over 40 counties in the region were declared disaster areas due to the storm [2]. At least 68 people died in Texas from the direct effects of Hurricane Harvey, representing the largest number of direct deaths from a tropical cyclone in the state since 1919 [1]. Less well understood, however, are the long term health and housing effects that will inevitably result from the storm.

Flooding in Houston brings additional environmental health concerns to its residents. The greater Houston area includes roughly 570 chemical plants, 43 Superfund sites (13 of which flooded), 9 refineries, 188 cement batch plants, 80 metal recycling facilities, as well as numerous underground storage tanks [3–6]. For the three most prevalent carcinogenic hazardous air pollutants—benzene, 1,3 butadiene, and formaldehyde – the area is subject to routine industrial emissions of over 472 tons/year [7]. Industry reported unexpected emissions events during the week of Harvey, releasing ~30 tons of benzene and 34 tons of 1,3 butadiene. Events at the Arkema plant in Crosby required evacuation of the residents in that area [8]. Elevated levels of benzene were observed in the Manchester neighborhood near the Houston Ship Channel with measurements of over 300 ppb, exceeding the 100 ppb level at which special breathing equipment is recommended [9, 10]. Measurements by EPA of dioxin in the San Jacinto riverbed were 70,000 nanograms per kilogram, compared to the recommended clean up level of 30 nanograms per kilogram for the site [11, 12].

Receding flood waters resulted in widespread mold and bacterial contamination in residential and commercial structures [13]. There is also uncertainty related to the complex mixtures of contaminants, as well as the impact of psychological stress. The potential for health risk is clear.

Public health registries play a key role in our understanding of health outcomes resulting from exposure to an event, disaster or hazardous agent. Registries collect and maintain data to facilitate actionable research, monitor health outcomes in exposed populations, and serve as a communication channel to provide valuable information and resources to affected communities. Events that have prompted the creation of registries include the atomic bombings of Hiroshima and Nagasaki, the Three Mile Island accident, the Chernobyl disaster, the Oklahoma City Bombing, and the 2001 attacks on the World Trade Center [14–18]. The US Department of Veteran Affairs and Department of Defense also maintain several registries to monitor the health of veterans exposed to hazardous substances during military service [19].

The World Trade Center Health Registry (WTCHR) is the largest registry in the US to track the health effects of a disaster or exposure and is perhaps the most well-known of all the registries [20, 21]. The WTCHR has been instrumental in our understanding of the long-term health outcomes of 9/11 including causal relationships between exposure to toxic smoke, dust, and debris and long-term health outcomes like asthma and other lung diseases [22]. In addition, the WTCHR has identified long-term mental health impacts including post-traumatic stress disorder (PTSD) [23, 24]. These findings helped create new policies that provide medical benefits to individuals directly impacted by the attacks [25]. The WTCHR also informs clinical guidelines and planning for future emergencies.

The scope and scale of Harvey, along with the clear lessons learned from the WTCHR, called for an innovative approach to understanding the environmental health and housing risks in the aftermath of the storm. With advice and guidance from New York colleagues, we launched the Hurricane Harvey Registry, a community health and housing registry, in April 2018. The registry is unique in its timeliness (e.g., the WTCHR was launched two years after 9/11) and start as a grassroots initiative between academia, local public health agencies, and community stakeholders. Often registries are managed through central government agencies, and the time between event or exposure and the start of data collection is too long to capture short-term effects [26]. Because of the flexibility built into the backend of the Hurricane Harvey Registry, we were able to launch surveys related to the May 2019 and Tropical Storm Imelda (September 2019) flooding events within weeks. Because of our desire to be broadly responsive to storms and flooding events, we renamed the Hurricane Harvey Registry as the “Texas Flood Registry.”

We are using state of the art data and exposure science to identify who was (and continues to be) exposed to what and in so doing establish baseline understanding of the risks for longer term environmental health effects from storms. The “who” is characterized at the individual and population level. The “what” uses exposure science to characterize air-, soil- and water-based contaminant exposures, as well as indoor environmental exposures resulting from flooding. In addition, data collected has been used by local health departments to better understand how social factors can impact health. Here we provide an overview of our experiences in establishing the registry. In addition, we perform an example analysis that illustrates potential uses of registry data.

## Materials and methods

### Building partnerships

The Texas Flood Registry represents a collaboration among Chambers County Health Department, Corpus Christi-Nueces County Public Health District, the Environmental Defense Fund, Fort Bend County Health and Human Services, Harris County Public Health, Houston Health Department, Montgomery County Public Health, Victoria County Office of Emergency Management, the Kinder Institute Urban Data Platform at Rice University, and the Children’s Environmental Health Initiative, which was then at Rice University. Collaboration with county and municipal governments is key to the success of the registry. As professionals at the forefront of disaster response and recovery, our collaborators provide real-time information regarding who was impacted and how to best engage them in the registry. In addition, their awareness of emerging health issues from the storm informs data collection and analysis. Our collaborators use the research to action framework to develop tailored interventions on the basis of what we learn from the Registry.

Decisions about when and how to modify or extend the registry are made through a collaborative and consultative process. Representatives from each partner meet via videoconference. Initially these meetings happened on a weekly or bi-weekly basis, however, with processes now streamlined, meetings happen monthly. Decisions from this group are implemented by the technical staff.

### Outreach and recruitment

The TFR team employs a data-driven outreach strategy that involves engagement with the public through media, business, and community partnerships. Participants in the Texas Flood Registry primarily enroll through the Registry’s website (https://www.floodregistry.rice.edu). Of note, the entire Registry website and associated surveys are available in English and Spanish.

Early on, we encountered difficulties reaching community members who might not have access to personal computers or wireless connections and were not fluent in English. We responded by supplementing online recruitment with multiple access points for hard-to-reach populations. At in-person events, staff spoke directly with community members to share information about the registry in the context of larger community concerns. Spanish speaking team members enabled the registry to engage in transparent, culturally relevant outreach with Spanish speakers. Community members joined the registry by completing the initial core survey on tablets or paper forms. Registry staff provided paper surveys and prepaid envelopes to attendees who were unable to complete the survey during events. Community members also signed up to receive the survey link via text message. Through partnerships with elected officials and local organizations, paper surveys with prepaid envelopes and promotional materials with the website URL or QR code were also distributed at health clinics, community centers, libraries, and other spaces with public computers and Wi-Fi. Partnerships with local public health agencies have proven critical for buy-in from community stakeholders.

In addition, we developed an extensive portfolio of Public Service Announcements and print, radio, and television interviews for distribution in English and Spanish to diverse media outlets. The registry has successfully engaged local and national media through on-air interviews with entities like The Weather Channel and over $100,000 in in-kind promotional support from the Houston Chronicle, Houston Public Media, Liberman Broadcasting, Radio One, Telemundo, and Univision. In addition, famous Houstonians including Gerald Green of the Houston Rockets, former Houston Mayor Annise Parker, and musicians Paul Wall, Brian Courtney Wilson, and Uche have embraced the TFR as a critical tool for our community and recorded Public Service Announcements in support of the TFR.

Participants typically enrolled one or more months after flooding events due to the chaos introduced into people’s lives in the immediate aftermath of storms. Registrants indicate consent by selecting “I agree” on the online consent form or by signing the paper consent form. In addition to offering a mobile-ready and easy-to-use survey interface for completing surveys designed and deployed by the Registry’s technical team, the Registry’s website also includes resources for victims of past and recent flooding events, information on the partners that support the Registry, a FAQ about the registry, and a way to contact the Registry team.

### Technical infrastructure

The registry’s technical infrastructure was built from scratch using modern and open software and web development tools, rather than deploying an out-of-the-box solution (e.g., WordPress). While this approach increases complexity and maintenance, it also maximizes flexibility and security. Because the TFR team owns the source code of the registry, it can be easily and quickly modified as new capabilities become necessary. This has been made amply clear as we have successfully used the same technical backbone to deploy a COVID-19 registry.

The registry follows a conventional three-tier website architecture: [1] a frontend consisting of the webpages that users interact with; [2] a secure database that stores user and survey information; and [3] a backend that coordinates between the two and manages user sessions with the Registry. This architecture is deployed in the Amazon Cloud and is HIPAA-compliant, leveraging best practices and advice from the Amazon Cloud team.

### Survey content

The core survey included 45 questions on registrants’ experience during Harvey, broken down into sections covering background demographics, the impact of Hurricane Harvey on their living environment, their physical and mental health prior to, during and after the storm event, and an open ended response question. A checklist of potential physical symptoms of interest was created in collaboration with health departments to include (1) runny nose, cough, postnasal drip, itchy eyes, or dry/scaly skin, (2) headaches/migraines, (3) problem concentrating, (4) skin rash, and (5) shortness of breath, chest tightness or pain, whistling or wheezing sound when exhaling, coughing or wheezing attacks by the cold or the flu, or trouble sleeping because of these respiratory symptoms. To evaluate mental health, the survey implemented the 15-item self-report impact of event scale (IES) tool [27]. The full survey is presented in the Supplemental Material.

In early May 2019, a severe weather event hit parts of Texas, including the Greater Houston area. Torrential rain caused 37,000 power outages, at least three bayous to overflow their banks, and dozens to be reported trapped in floodwater on Interstate 10 [28]. In response to this event, the Texas Flood Registry added questions to its core online survey so that new registrants have the option to document how this flood event affected them. These additional questions are also available to previous registrants in an online supplemental survey. We responded similarly when Tropical Storm Imelda inundated areas along the Gulf Coast in September 2019. Data collection is ongoing, open to all who were present during the event to report their experiences and impacts from each storm.

### Statistical analysis

We have consolidated and spatially referenced the environmental data collected during and in the aftermath of the storms. Samples collected before, during, and after the storm are housed within the Urban Data Platform at Rice University and freely accessible. Multiple research teams collected extensive datasets that include sampling (E. coli, Volatile Organic Compounds (VOCs), Benzene/Toluene/Ethylbenzene/Xylenes (BTEX), fine particulate matter (PM2.5), ozone, and others), rain melt/precipitation and flood gage readings, drinking water quality, Federal Emergency Management Agency (FEMA) shelters, power outages, city requests for service, debris management sites, transportation infrastructure, FEMA Individual Assistance records, aerial imagery, and more. Additional information on how, when, and by whom these different data were collected is available in the metadata associated with each dataset stored on the Urban Data Platform. In addition, we have also linked via shared geography a host of environmental and social data that are also relevant to assessing the health and well-being of Houston area residents. All data are space- and time-referenced, allowing for integration with the registry data.

The Registry datasets and linked environmental and social data are stored on Rice’s Urban Data Platform, a secure data repository and computing environment that holds over 5Tb of geo-referenced curated data (see www.kinderudp.org). In addition to the secure platform, the Urban Data Platform supports a second, open platform through Amazon Cloud Services. This second platform allows registered users to download public research-ready geo-referenced data. Each curated data set housed on the Urban Data Platform is issued a permanent Digital Object Identifier (DOI) through Rice’s Fondren Library partnership with DataCite. Researchers may also contribute data to the UDP. In doing so, the data has a permanent home and receives a DOI for contributors and users to cite the data. The permanency represented by the DOI supports the scientific goal of reproducible research. User-contributed data are reviewed by the UDP oversight committee and senior data manager for quality assurance before being placed on the platform and receiving a DOI.

As an example of the kinds of statistical analysis that the registry enables, we performed a multivariable logistic regression at the individual level to determine the relationship between floodwater exposure and adverse health outcomes. Using data collected between April 2018 and March 2020, we fit separate models for each adverse health outcome with an indicator for self-reported flooding or skin contact with floodwater as the predictor variable. All models controlled for age, gender, race/ethnicity, education level, and self-reported health status.

We also performed a spatial analysis at the census tract level using a binomial Bayesian conditional autoregressive (CAR) spatial model with special consideration given to capture the localized spatial autocorrelation [29]. The City of Houston maintains a dataset of houses damaged during Harvey [30]. To conduct this analysis, we merged Registry data collected between April 2018 and April 2019 with the damage assessment dataset and then estimated the spatial structure at the census tract level. We used the damage assessment data to calculate the percentage of damaged housing units in each Census tract. For tracts with missing data, we used Bayesian spatial smoothing to estimate housing damage based on data from adjacent areal units. An indicator variable for reporting at least one adverse health effect served as the dependent variable. Explanatory variables included census tract level unemployment rate, percentage of damaged housing units, percentage of people receiving public assistance, median household income, and racial isolation.

Finally, we analyzed trends in rainfall for the region using a 40 year-temporal windowed generalized Pareto peak over threshold model fit to the history for each rain gages located throughout the region—data also available through the UDP [31].

The work of the Hurricane Harvey Registry, as well as the eventual transformation to the Texas Flood Registry, were all undertaken under the auspices of a human subjects research protocol approved by the Rice University Institutional Review Board (IRB-FY2018-95).

## Results

**Fig. 1:**
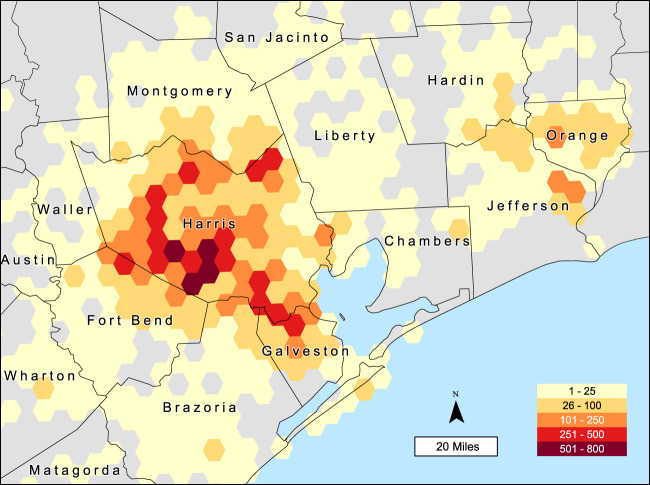
Geographic distribution of TFR registrants. Number of respondents per 20 square mile hexagons as of March 31, 2020.

As of March 31, 2020, we have enrolled 20,067 individuals in the registry, with wide geographic coverage (see Fig. 1). Of these registrants, 19,993 have completed the initial core survey on Hurricane Harvey. Initially, our web-based design introduced selection bias to the registry that led to an under-representation of minority community members. Our current tailored recruitment strategies are resulting in new registrants that are more representative of the area demographics. We create quarterly analysis-ready TFR curated datasets used by our health department partners to understand impacts and design interventions.

Registrants completing the initial core survey between April 2018 and March 31, 2020 were asked to indicate whether they had any of the symptoms in Table 1 in the year following Hurricane Harvey. Multivariable logistic regression shows that respondents whose home flooded had increased odds of adverse health effects than those who did not flood (*p* value < 0.0001). In a model adjusted for age, gender, race, education level, and self-reported health status, the odds of runny nose, itchy eyes, and dry skin is 45% greater for those whose homes flooded than those whose homes did not. Similarly, the odds of headaches/migraines and respiratory symptoms including shortness of breath are more than 60% greater for those whose homes flooded than those whose homes did not. The odds of skin rash and problems concentrating are more than two times higher for those whose homes flooded.

**Table 1:** Multivariable logistic regression for home flooding and health effects reported by HHR registrants (*N* = 18,787).

Odds ratio estimates															
	Shortness of breath			Skin rash			Problem concentrating			Headaches/migraines			Runny nose/itchy eyes/dry skin		
Effect	Point estimate	95% Wald		Point estimate	95% Wald		Point estimate	95% Wald		Point estimate	95% Wald		Point estimate	95% Wald	
		Confidence limits			Confidence limits			Confidence limits			Confidence limits			Confidence limits	
Flood water exposure															
Home flooded	1.63	1.51	1.75	2.27	2.05	2.50	2.66	2.47	2.86	1.64	1.54	1.76	1.45	1.36	1.54
No flooding															
Gender															
Male	0.70	0.64	0.77	0.97	0.86	1.09	0.60	0.55	0.66	0.44	0.40	0.49	0.67	0.63	0.72
Female															
Race/ethnicity															
NH Other	1.79	1.44	2.21	1.91	1.47	2.48	1.25	1.00	1.56	1.85	1.51	2.26	1.32	1.08	1.60
NH Asian	0.74	0.52	1.04	1.28	0.87	1.88	0.56	0.40	0.78	0.65	0.48	0.88	0.60	0.47	0.76
Hispanic	1.00	0.90	1.11	1.45	1.27	1.65	0.98	0.88	1.08	1.06	0.97	1.17	0.81	0.75	0.89
NH Black	1.26	1.10	1.44	1.45	1.23	1.72	0.79	0.68	0.90	1.17	1.04	1.33	0.80	0.71	0.90
NH White															
Education group															
High school graduate or less	1.22	1.10	1.37	0.96	0.82	1.11	0.73	0.65	0.81	1.13	1.02	1.26	0.86	0.78	0.94
Some college or associates	1.42	1.31	1.54	1.25	1.12	1.39	1.04	0.96	1.12	1.28	1.18	1.37	1.18	1.10	1.26
Bachelors or higher															
Age group															
81+	3.44	1.79	6.59	1.01	0.41	2.48	0.92	0.50	1.70	0.32	0.17	0.64	1.42	0.89	2.28
61–80	2.63	1.51	4.58	1.43	0.71	2.86	1.08	0.67	1.75	0.76	0.49	1.16	1.22	0.85	1.74
41–60	2.27	1.31	3.94	1.66	0.83	3.30	1.35	0.84	2.17	1.39	0.91	2.12	1.20	0.84	1.71
21–40	1.70	0.98	2.96	1.27	0.63	2.53	0.99	0.61	1.59	1.62	1.06	2.48	1.25	0.88	1.79
18–20															
Health self-assessment															
Excellent	0.14	0.12	0.18	0.24	0.18	0.30	0.24	0.20	0.30	0.25	0.20	0.30	0.30	0.25	0.36
Very Good	0.18	0.15	0.21	0.27	0.22	0.33	0.33	0.28	0.39	0.31	0.26	0.36	0.41	0.35	0.49
Good	0.28	0.23	0.32	0.36	0.30	0.44	0.41	0.35	0.48	0.42	0.36	0.49	0.52	0.44	0.61
Fair	0.51	0.43	0.60	0.51	0.42	0.62	0.59	0.50	0.70	0.63	0.53	0.74	0.71	0.60	0.84
Poor															

Differences in reported symptoms are even more substantial when we compare respondents whose skin came into contact with floodwater with those whose skin did not come into contact with water (Table 2). The odds of having headaches/migraines and runny nose, itchy eyes, and dry skin are more than 70% greater for those whose skin came into contact with floodwater. Respondents whose skin came into contact with floodwater were also 82% more likely to report shortness of breath. The odds of skin rash and problems concentrating are 3.5 and 2.5 times higher, respectively, for respondents whose skin came into contact with floodwater.

**Table 2:** Multivariable logistic regression for skin contact with floodwater and health effects reported by HHR registrants (*N* = 18828).

Odds ratio estimates															
	Shortness of breath			Skin rash			Problem concentrating			Headaches/migraines			Runny nose/itchy eyes/dry skin		
Effect	Point estimate	95% Wald		Point estimate	95% Wald		Point estimate	95% Wald		Point estimate	95% Wald		Point estimate	95% Wald	
		Confidence limits			Confidence limits			Confidence limits			Confidence limits			Confidence limits	
Flood water exposure															
Skin contact with flood water	1.82	1.69	1.97	3.54	3.12	4.00	2.54	2.35	2.75	1.76	1.64	1.89	1.73	1.62	1.83
No contact with flood water															
Gender															
Male	0.66	0.60	0.72	0.86	0.76	0.97	0.55	0.50	0.60	0.42	0.38	0.46	0.63	0.59	0.68
Female															
Race/ethnicity															
NH Other	1.74	1.40	2.15	1.83	1.41	2.38	1.20	0.97	1.49	1.81	1.47	2.21	1.29	1.06	1.57
NH Asian	0.80	0.57	1.13	1.49	1.01	2.19	0.65	0.46	0.91	0.71	0.52	0.96	0.65	0.51	0.82
Hispanic	1.03	0.93	1.15	1.54	1.35	1.76	1.05	0.95	1.16	1.10	1.00	1.21	0.84	0.77	0.91
NH Black	1.39	1.22	1.58	1.73	1.46	2.05	0.92	0.80	1.06	1.29	1.14	1.46	0.87	0.78	0.98
NH White															
Education group															
High school graduate or less	1.22	1.10	1.36	0.95	0.81	1.10	0.77	0.69	0.86	1.14	1.03	1.27	0.84	0.77	0.93
Some college or associates	1.41	1.31	1.53	1.23	1.10	1.37	1.05	0.97	1.14	1.27	1.18	1.37	1.16	1.09	1.24
Bachelors or higher															
Age group															
81+	4.45	2.32	8.53	1.53	0.62	3.77	1.50	0.82	2.76	0.40	0.21	0.79	1.77	1.10	2.85
61–80	3.01	1.73	5.23	1.77	0.88	3.55	1.41	0.88	2.27	0.83	0.55	1.27	1.37	0.96	1.97
41–60	2.44	1.41	4.23	1.85	0.92	3.68	1.60	1.00	2.56	1.44	0.95	2.18	1.27	0.89	1.82
21–40	1.80	1.03	3.12	1.36	0.68	2.73	1.12	0.70	1.81	1.65	1.09	2.51	1.33	0.93	1.89
18–20															
Health self-assessment															
Excellent	0.14	0.12	0.17	0.23	0.18	0.30	0.24	0.20	0.29	0.24	0.20	0.29	0.30	0.25	0.36
Very Good	0.18	0.15	0.21	0.26	0.21	0.32	0.32	0.27	0.38	0.30	0.25	0.35	0.41	0.34	0.48
Good	0.27	0.23	0.31	0.34	0.29	0.42	0.39	0.33	0.46	0.40	0.34	0.47	0.51	0.43	0.60
Fair	0.50	0.42	0.59	0.49	0.40	0.60	0.57	0.48	0.67	0.60	0.51	0.72	0.70	0.59	0.83
Poor															

Differential effects were also observed among the control variables. Male gender and increasing self-reported health status have a protective effect. Conversely, we observe increased odds of adverse health outcomes with lower educational attainment. Older age was also associated with increased odds of shortness of breath. Non-white respondents were more likely to report shortness of breath, skin rash, and headaches but not problems concentrating or general allergic response (runny nose, itchy eyes, and dry skin).

In the census track analysis using Bayesian CAR methods, the percentage of homes flooded (95%) Credible Interval on the coefficients of the covariates (CI) (0.82, 1.1) was associated with respondents reporting one or more adverse health effects from Harvey. Furthermore, median household income was inversely related to adverse health effects (95% CI (−0.18, −0.08)). An additional take-away from this analysis is that the spatial component is important to any modeling we consider (95% CI for spatial coefficient (0.003, 0.066)).

**Fig. 2:**
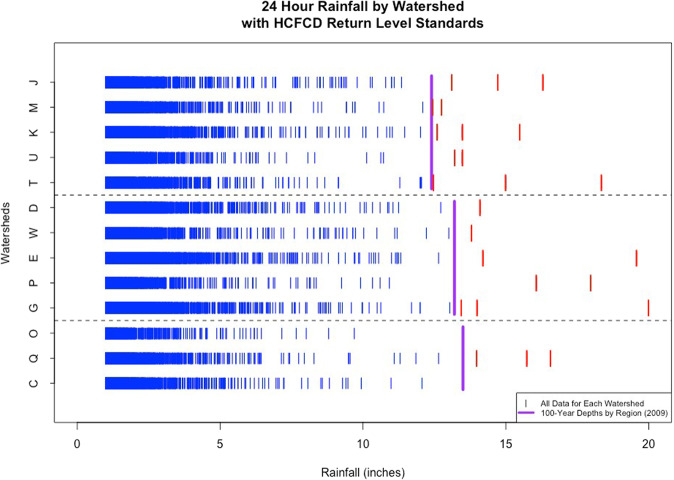
24-h rainfall by watershed and grouped by region in Harris county. Purple vertical lines represent the 2018 Harris County Flood Control District standards for each region. Individual vertical lines represent 24-h observed rainfall event, with blue indicating the event is below the design level and red indicating it is above. Only 24-h rainfall events greater than 1 inch are plotted.

In addition to analysis of the registry data, we have identified a trend in rainfall for the region. This has led to a reconfiguration of regional flood models (see https://www.sspeed.rice.edu/harvey-projects) [32]. Figure 2 depicts 24 h rainfall events from representative gages located in the watersheds (denoted by letters) for the area. A map of the respective watersheds and their regions, as depicted in Fig. 2, is available in Fagnant et al. [31]. Several gages have rainfall levels spanning 100 years, however most span 30–60 years. The 2018 engineering 100 year return level design value is included in the figure, depicted by vertical purple bar for each region. The 100 year return level represents the amount of rainfall expected to be met or exceeded on average once every 100 years, or the rainfall level associated with an expected 1% chance of flooding per year. The observed rainfall measurements above the 2018 design levels are highlighted in red. A critical issue is the large number of red values above the 100 year return levels, as well as the extent of the excess (e.g., 20 inches of rain in watershed G).

**Fig. 3:**
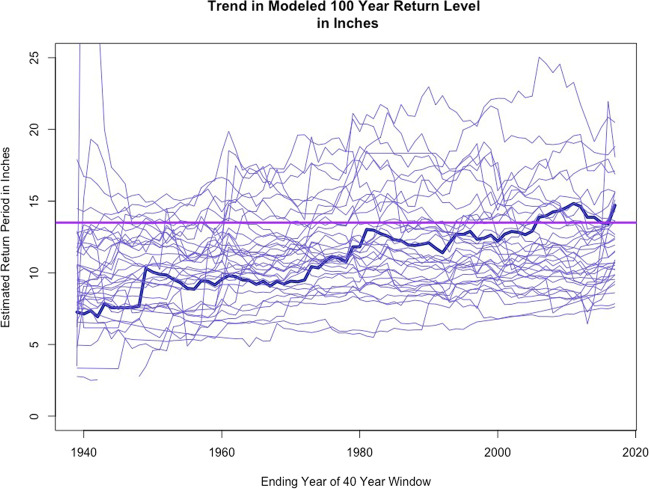
Trend in modeled 100 year return level in inches. Each time series represents the modeled 100 year 24 hour rainfall event return level based on a 40 year-temporal sliding window with the generalized Pareto peak over threshold model fit to each gage with at least an 80 year record in the region. A map of all monitors in the region is available in Fagnant et al. [31]. The horizontal line represents the stationary 100 year 24 hour return rainfall design level for the region.

To ascertain whether trends are present in the observational data, a 40 year-temporal windowed generalized Pareto peak over threshold model was fit to the history for each gage. The modeled 100 year return levels for all available gages are presented in Fig. 3. The estimated return levels are key inputs to the extensive flood modeling taking place for the Houston area [33]. These new maps are helping us to identify those areas and individuals who are most vulnerable to future flooding events.

## Discussion

The Texas Flood Registry (TFR) is the first registry of its kind to track the health and housing impacts of a natural disaster. The TFR is unique in that it is a local initiative that came together quickly in the aftermath of Hurricane Harvey and has been rapidly deployed following more recent storms, collecting data on both the short- and long-term effects of flood events. The Texas Flood Registry has created new opportunities for collaboration between academic institutions, public health agencies, and community stakeholders in the region. The boots on the ground knowledge of local health departments and community partners helps to identify key areas for future environmental health research. Furthermore, engaging the academic community in research and public health surveillance promotes knowledge development that informs long-term recovery and planning for future disasters.

The UDP makes it possible for any researcher interested in the long-term effects of flooding events to contribute their own data and tap into the extensive data resources developed by the registry. A mixed methods approach to recruitment and calls to action that ask community members to join the registry whether impacted severely, lightly, or not at all help to create a registry population that represents a broad range of experiences. In addition, rather than relying solely on self-reported exposure information, the registry integrates space- and time-referenced environmental and socio-demographic data to characterize exposures before, during, and after an event, reducing the potential for misclassification and self-report bias.

We have published an initial report on findings from the registry, which received wide media coverage (see https://www.youtube.com/watch?v=FqlraoOZuLA). On September 4, 2019, the registry convened a congressional briefing on the campus of Rice University. The briefing was used as an opportunity to familiarize state legislative staff with the purpose and importance of the registry, how the registry benefits the state, and how members of the Texas Legislature can support the registry. The briefing included a presentation of the latest findings, panel discussion with health department collaborators, and question-and-answer session. The event was open to the public and also broadcast via live stream.

In addition, our quarterly analysis-ready TFR curated datasets are used by our health department partners to understand impacts and design interventions. For example, immunocompromised patients are at greater risk of invasive mold infections (IMI), and exposure to high levels of mold following flooding may increase the risk of disease. However, since IMI is not a notifiable condition, data to understand the health impact of flooding events is limited. Harris County Public Health and the Houston Health Department are using the TFR in a joint project to determine the invasive mold infection incidence pre- and post-Hurricane Harvey, as well as to identify whether molds known to cause IMI are present in flooded homes. The Houston Health Department is also using the TFR to discern the relationship between Hurricane Harvey and asthma exacerbation among Medicaid and CHIP beneficiaries (children < 18) residing in 24 flood inundated Houston ZIP codes who used the emergency department before or after the event.

Global climate change may increase the number and strength of hurricanes hitting the United States and internationally and may increase flooding from non-hurricane weather events. Climate influences the frequency and intensity of storms, including hurricanes. Global climate science predicts higher storm surge levels, increased hurricane rainfall rates, increased hurricane intensity, and a higher proportion of hurricanes that will reach very intense (Category 4 and 5) levels in the future [34, 35]. In addition, stagnant weather patterns, considered by some to be a result of climate change, mean that storm rainfall and winds will remain in one area for longer than usual [36]—this phenomenon was certainly present when Hurricane Dorian moved very slowly and then essentially stalled over Grand Bahama Island in the Bahamas [37]. Even below-hurricane strength weather can result in significant rainfall and flooding as evidenced by the May 2019 storms in the Houston area [28] and the extensive flooding along the Missouri and Mississippi Rivers in spring 2019 [38].

Hurricanes and major flooding events commonly cause significant water-related damage that leads to short, medium, and long term health effects—and these effects are often concentrated in low income and minority communities. Low income and minority populations, as well as those with pre-existing health conditions, are often characterized as having increased factors for susceptibility and vulnerability to disease. Of those, the elderly of low income are reported to be most vulnerable and slowest to recover from disasters such as hurricanes [39]. In addition, poverty makes people more vulnerable to many adverse health outcomes associated with weather-related events [40–42]. A wide range of human health effects associated with extreme weather events such as hurricanes and flooding result from exposures associated with the release of toxic chemicals from landfills, contamination of drinking water with raw sewage as a result of damage to water infrastructure, increased concentrations of air pollutants that are especially harmful to susceptible populations such as children, the elderly, and those with asthma or cardiovascular disease, and myriad other hazards [43].

Our findings are consistent with the literature and anecdotal reports being offered by local physicians, suggesting home flooding and skin contact with water during Hurricane Harvey adversely impacted physical health in the year following the storm.

Population-level data is imperative to understanding the downstream health effects of these major events. Increases in the incidence and intensity of extreme weather events such as hurricanes and floods may adversely affect people’s health immediately during the event or in the aftermath of the event [43]. A key element to mitigating potential adverse health outcomes from these weather events is a better understanding of diseases and the unique risks of exposed populations. As such, rapid collection of data, as well as ongoing monitoring, is essential to understanding downstream health effects, crafting tailored and specific interventions, and improving the capabilities of health and emergency services to address future disasters. Previous registries developed post-disaster have illustrated the importance of such information to public health protection, as well as in planning for future disaster events [22]. The TFR data are and will continue to be used to inform strategies and interventions aimed at addressing health risks.

We intentionally chose a structure for the TFR and the UDP that make it possible for any researcher interested in the relationship between flooding events and health effects to tap into the extensive data resources developed. We welcome inquiries and collaborations [44].

The underlying technical backbone to the registry was designed to flexibly adapt to any type of disaster, and we successfully deployed it for the May 2019 and Tropical Storm Imelda flooding events. To deploy surveys for other public health disasters, our tool organizes individual storms or events as separate modules in the database and allows for the easy addition of new modules and multiple surveys. As evidence of its wider flexibility, the same technical backbone is now being deployed to support a COVID-19 registry, specifically designed to support local health departments in planning their response and interventions related to the global pandemic. The tool was adapted to allow surveys to be completed more than once for collecting follow-up and longitudinal data, which was vital to monitoring the ever-changing nature of the pandemic.

Our combined data and exposure science approach gives us a comprehensive understanding of the environmental health risks created by Hurricane Harvey and other major flooding events. The combination of our registry including flooded and non-flooded residents in the greater Houston area with a comprehensive assessment of the potential exposures residents faced (and continue to face) provides an unprecedented opportunity to evaluate the environmental health risks created by major storm events, as well as the effectiveness of interventions put into place in response to the storm. Houston is the most diverse city in the country, with the demographics that we expect the rest of the country to have in ~2050. Lessons learned in Houston can help cities nationally and internationally in preventive intervention, planning, and response.

## Supplementary information

Supplementary information
