# Supplementary material for: The Texas flood registry: a flexible tool for environmental and public health practitioners and researchers
Source: J Expo Sci Environ Epidemiol. 2021 Jun 26;31(5):823–31. doi: 10.1038/s41370-021-00347-z (PMC8234769; doi:10.1038/s41370-021-00347-z)
Supplement: Supplementary file 1 — Supplementary information [file 41370_2021_347_MOESM1_ESM.pdf]

Hurricane Harvey Registry

The Hurricane Harvey Registry collects information on the impact of Hurricane Harvey on our Greater Houston area community.

Participants take on average about 10 minutes to complete this survey.

What is included in the Registry?

A brief core survey with questions on health, housing, and vehicle damage resulting from Hurricane Harvey.

Why should I take the Registry's survey?

The information gathered through the Registry will be used to serve and inform the Greater Houston area community.

Measuring who is being impacted and how will help to direct the continuing Hurricane Harvey recovery efforts, and plan for the future.

Who should complete the Registry's survey?

Any adult in the Harvey-impacted, Greater Houston area.

If there is more than one adult per household, we ask that each adult complete the survey individually.

Your answers are important to us whether Harvey impacted you severely, lightly, or not at all.

The Hurricane Harvey Registry is a joint venture of the Environmental Defense Fund, Fort Bend County Health Department, Harris County Public Health, Houston Health Department, Montgomery County Health Department, and Rice University. It is funded by the Environmental Defense Fund, the Cullen Trust for Healthcare, and the National Institutes of Health.

Consent Form

Purpose of this Registry

The Hurricane Harvey Registry will collect health, location, and exposure information from Houston area residents. This information can help identify health trends and risks associated with Hurricane Harvey. The registry will also support planning for future natural disasters and policy making by city officials. This project is led by Dr. Marie Lynn Miranda from Rice University. This project is funded by the National Institutes of Health, the Cullen Trust for Healthcare, and the Environmental Defense Fund. Please read this consent form carefully and take your time deciding whether to participate.

Procedures / How the Survey Works

If you choose to participate, you will be asked to complete a paper survey with questions about you and your experience before, during, and after Hurricane Harvey. Participating in this study is completely voluntary and you may end your participation at any time. Most of the survey questions can be skipped if you do not want to answer them. The survey will take approximately 10 minutes. Follow up surveys will be available, but optional. You must be 18 or older to participate in this study.

Confidentiality

When you return this survey to the Harvey Registry team, it will be placed in a secure lock box and transported to Rice University. Data from this survey will be scanned and stored in a secure electronic system by IT experts from Rice University. This survey will be stored in a secure, access-controlled location at Rice University. Results of this study will be published, but will not include any information that would identify you or any other participant.

Data from this survey may be made available to other researchers outside of the Harvey Registry team. If researchers wish to use your information, they must have approval from Rice University and their home organization. They will also need to show how their research could benefit the greater Houston community. No one will see your data that does not have permission to see it.

Risks and Benefits

The risks from participating in this study are minimal. However, some questions will ask about your location, activity, and experiences during and following Hurricane Harvey. For some participants, this may be distressing. If you do feel distressed, free 24/7 counseling and support is available from the Red Cross Distress hotline at 1-800-985-5990, or by texting "TalkWithUs" to 66746.

While you may not benefit personally from filling out a questionnaire, the information in the registry will help us understand the health and housing impacts of Hurricane Harvey on the greater Houston area. You may receive reports from our team that tell you what we are learning from the registry. The reports will also be shared with government officials to guide the response to Harvey and plan for Houston's future.

Problems, Questions, or Concerns

For questions about the study, or if you have complaints, concerns or suggestions, you may contact Dr. Marie Lynn Miranda or Rashida Callender at harveyregistry@rice.edu. For questions about your rights as a research participant, or to discuss problems, concerns or suggestions related to the research, or to obtain information or offer input about the research, you should contact Stephanie Thomas, Compliance Administrator, at Rice University. Email: irb@rice.edu or Telephone: 713-348-3586. Please reference Rice IRB Number IRB-FY2018-95.

Statement of Consent

"The purpose of this study, procedures to be followed, risks and benefits have been explained to me. I understand that I can contact Dr. Marie Lynn Miranda or Rashida Callender to discuss problems, concerns, or questions, or to obtain more information. I have read this consent form and, by completing and returning a survey, agree to be in this study, with the understanding that I may withdraw myself at any time."

______________________________ ______________________________ __________

Signature Printed Name Date

Registration

We ask the following questions to create a record for you in the Hurricane Harvey Registry. Creating a record is necessary for your survey answers to be saved. If you agree to be re-contacted and provide your contact information, you may be notified about news and resources that could be relevant to you.

Required questions are marked with an asterisk (*). Some questions are optional, but please answer as many as you are comfortable with. Your answers help to serve and strengthen our greater Houston community.

1. First Name *: ______________________________
2. Middle Name: ______________________________
3. Last Name *: ______________________________
4. Date of Birth (mm/dd/yyyy) *: ________ / ________ / ________
5. Additional surveys may help us to better understand the impacts of Harvey, and new resources for those affected by Harvey may become available. Do you agree to be re-contacted for either of these reasons? *

❍ Yes

❍ No

6. Contact E-mail : ______________________________
7. Phone Number: ______________________________
8. Current mailing address:

Address: ______________________________

Apartment or Suite: ______________________

City: ______________________________

State: ______________ Zip: ___________

Survey

Impact of Harvey – Living Environment

We will now ask you questions about your living environment before and after Hurricane Harvey.

9. How many people (including yourself) lived in your household at the time of Hurricane Harvey? : __________

10. Of these, how many (including yourself) were:
    a. Children under 18 years old: __________
    b. Adults between 18-59 years old: __________
    c. Seniors (60+): __________
    d. Disabled: __________
    e. Pregnant women: __________

11. For the people in your household, select the answer that best describes your experience.
- ❍ Members of my household continued living together during Hurricane Harvey.
- ❍ Members of my household were temporarily separated by Hurricane Harvey but are all back together now.
- ❍ Members of my household were temporarily separated by Hurricane Harvey and are still separated.
- ❍ Members of my household were permanently separated due to Hurricane Harvey.

12. Were any vehicles owned by a person in your household damaged or lost because of Hurricane Harvey?
- ❍ Yes
    - o How many? __________
- ❍ No

13. Did you or any member of your household lose income as a direct consequence of the hurricane? *
- ❍ Yes
- ❍ No

14. Where did you live **before** Hurricane Harvey?
- ❍ I lived in a home that I owned
- ❍ I lived in a home or apartment that I rented
- ❍ I lived with family/friends
- ❍ I lived in a temporary shelter
- ❍ I was homeless (**IF YOU ANSWERED "HOMELESS", SKIP TO QUESTION 31)**
- ❍ Other (describe): ____

15. This home was a/an:
- ❍ Apartment
- ❍ Condominium
- ❍ Hotel/Motel
- ❍ Mobile Home / Trailer
- ❍ Single Family Dwelling
- ❍ Other (describe): __________________________

16. Your home address when Hurricane Harvey hit * :

Address: ___________________________________

Apartment or Suite: __________________________

City: ______________________________________

State: __________________ Zip: ______________

17. Was your home built before 1970?

- ❍ Yes
- ❍ No
- ❍ Don't know

18. Did you have to leave your home because of Hurricane Harvey? *

- ❍ Yes
- ❍ No (**<u>IF YOU ANSWERED "NO", SKIP TO QUESTION 21)</u>**

19. If you had to leave your home because of Harvey, where did you go?

- ❍ I was homeless
- ❍ I lived in a temporary shelter
  Please indicate which one(s) below:
  - ❑ Houston: Chinese Community Center
  - ❑ Houston: Copperfield Church
  - ❑ Houston: George R. Brown Convention Center
  - ❑ Houston: Greenspoint Mall Center
  - ❑ Houston: Houston Community College
  - ❑ Houston: NRG Center
  - ❑ Houston: Toyota Center
  - ❑ Port Arthur: Thomas Jefferson Middle School
  - ❑ Port Arthur: Bob Bowers Civic Center
  - ❑ Beaumont: Sterling Pruitt Center
  - ❑ Dallas: Kay Bailey Hutchison Convention Center
  - ❑ Austin: Met Center
  - ❑ Other (describe): __________________
- ❍ I lived with family or friends
- ❍ I lived in a mobile home or trailer
- ❍ I lived in a hotel or motel
- ❍ I lived in an apartment or condominium
- ❍ I lived in a Single Family Dwelling
- ❍ Other: __________________________________

20. Have you moved back to the home you were living in before Hurricane Harvey?

- ❍ Yes
  - ○ Please indicate date you moved back (mm/dd/yyyy): __________ / ___________ / __________
- ❍ No

21. Did your home flood? *

❍ Yes

❍ No (**<u>IF YOU ANSWERED "NO", SKIP TO QUESTION 25)</u>**

22. How long did it take for the flood water to recede?

____________ days

____________ hours (if less than 1 day)

23. How high was the flood water level in your home?

❍ Not sure

❍ Less than 1 inch

❍ Between 1 inch and less than 2 inches

❍ Between 2 inches and less than 3 inches

❍ Between 3 inches and less than 12 inches

❍ Between 12 inches and less than 14 inches

❍ Between 14 inches and less than 18 inches

❍ Between 18 inches and less than 24 inches (2 feet)

❍ 2 feet or more (Please provide the water level in feet): ____________

24. Did the water level reach the bottom of any of your electrical outlets?

❍ Yes

❍ No

❍ Not sure

25. Was your home damaged because of Hurricane Harvey?*

❍ Yes

❍ No (**<u>IF YOU ANSWERED "NO", SKIP TO QUESTION 28)</u>**

26. What types of clean up did you or someone else have to do in your home because of the rain or damage from Hurricane Harvey:

❑ Removing standing water and/or mud

❑ Removing carpets and/or floors

❑ Removing drywall, sheetrock, or wet insulation

❑ Removing baseboards, cabinets, doors, door trims, or window sills

❑ Replacing electrical outlets or switches

❑ Foam cleaning

❑ Pressure washing

❑ Using drying equipment (fan, dehumidifier, etc.)

❑ Other mold treatment and prevention measures

❑ Other (describe): __________________________

27. Were you in your home when these actions were taken?

❍ Yes

❍ No

28. Did your home lose electrical power?

❍ Yes

- For how long? Please indicate either number of hours: __________OR number of days: __________

❍ No

29. Were other homes on your block flooded?

❍ Yes

❍ No

❍ Don't know

30. Were there piles of trash / debris on your block?

❍ Yes

- How long did it take for these piles of trash / debris to be cleared? _____ weeks

❍ No

❍ Don't know

Impact of Harvey – Health

We will now ask you questions about your physical and emotional health.

31. What is your height?: ______ feet _____ inches How much do you weigh?: _____ pounds

32. Are you currently a tobacco user (including smokeless tobacco and e-cigarettes)?
- ❍ Yes
- ❍ No

33. Compared to other persons your age, would you say your health is…
- ❍ Excellent
- ❍ Very good
- ❍ Good
- ❍ Fair
- ❍ Poor

34. Has a health professional ever told you that you had:

Medical condition	Yes	No	Don't remember
**Heart disease**	❍	❍	❍
**Hypertension**	❍	❍	❍
**Diabetes**	❍	❍	❍
**Asthma or other lung disease**	❍	❍	❍
**Kidney disease**	❍	❍	❍
**A physical disability**	❍	❍	❍
**Cancer**	❍	❍	❍

35. Was your skin ever in contact with flood water during or right after the hurricane? *
- ❍ Yes
- ❍ No

36. Were you injured as a result of Hurricane Harvey?
- ❍ Yes
  - Please tell us more about this injury: ____
- ❍ No

37. Did you become ill as a result of Hurricane Harvey?
- ❍ Yes
  - Please tell us more about this illness: ____
- ❍ No

38. Were you hospitalized as a result of the hurricane? *
- ❍ Yes
  - For how long? __________ weeks OR __________ days
  - Why? ____
- ❍ No

39. Did you have any of the following symptoms in the year following Hurricane Harvey? Please indicate when these symptoms occurred. If you had these symptoms before Hurricane Harvey, please indicate if they were worse after the hurricane. Check all that apply.

	Before Harvey (before Aug. 25, 2017)	During and for the month following Harvey (Aug 25 – Sept 30, 2017)	1 to 6 months after Harvey (Oct 1, 2017 – Feb 28, 2018)	7 to 12 months after Harvey (March 1 – Aug 31, 2018)	Symptoms were worse than before Harvey
**Runny nose, cough, postnasal drip, itchy eyes, or dry/scaly skin**	❑	❑	❑	❑	❑
**Headaches / migraines**	❑	❑	❑	❑	❑
**Problem concentrating**	❑	❑	❑	❑	❑
**Skin rash**	❑	❑	❑	❑	❑
**Shortness of breath, chest tightness or pain, whistling or wheezing sound when exhaling, coughing or wheezing attacks by the cold or the flu, or trouble sleeping because of these respiratory symptoms**	❑	❑	❑	❑	❑
**None of the above** ❑					

40. Below is a list of difficulties people sometimes have after stressful life events. Please read each item, and then indicate how distressing each difficulty has been for you **DURING THE PAST SEVEN DAYS with respect to Hurricane Harvey**, which occurred on August 17, 2017 – September 2, 2017. How much were you distressed or bothered by these difficulties?

	Not at all	Rarely	Sometimes	Often
**I thought about it when I didn't mean to.**	❍	❍	❍	❍
**I avoided letting myself get upset when I thought about it or was reminded of it.**	❍	❍	❍	❍
**I tried to remove it from memory.**	❍	❍	❍	❍
**I had trouble falling asleep or staying asleep, because of pictures or thoughts about it that came into my mind.**	❍	❍	❍	❍
**I had waves of strong feelings about it.**	❍	❍	❍	❍
**I had dreams about it.**	❍	❍	❍	❍
**I stayed away from reminders of it.**	❍	❍	❍	❍
**I felt as if it hadn't happened or it wasn't real.**	❍	❍	❍	❍
**I tried not to talk about it.**	❍	❍	❍	❍
**Pictures about it popped into my mind.**	❍	❍	❍	❍
**Other things kept making me think about it.**	❍	❍	❍	❍
**I was aware that I still had a lot of feelings about it, but I didn't deal with them.**	❍	❍	❍	❍
**I tried not to think about it.**	❍	❍	❍	❍
**Any reminder brought back feelings about it.**	❍	❍	❍	❍
**My feelings about it were kind of numb.**	❍	❍	❍	❍

Demographic Information

Please tell us a little bit about yourself.

41. Choose the answer that best describes your educational background.

❍ I completed 8th grade
❍ I obtained a high school diploma
❍ I obtained a GED
❍ I attended some college
❍ I obtained an Associate's degree
❍ I obtained a Bachelor's degree
❍ I obtained a graduate degree (Master's, PhD)

42. What is your race? (Check one or more boxes.) *

❑ White
❑ Black/African American
❑ American Indian/Alaska Native
❑ Asian
❑ Native Hawaiian/Pacific Islander
❑ Some other race: ____

43. Are you of Hispanic, Latino, or Spanish origin? *

❍ Yes
❍ No

44. What is your gender? *:

❍ Male
❍ Female
❍ Other

Additional Comments

45. Has Hurricane Harvey affected your living environment or your health in other ways? Please let us know by writing in the box below.

____

____

____

You have reached the end of the Hurricane Harvey Registry survey! Thank you for your participation in this very important research project on the health of our communities.

Please invite others in your community to take the survey.

In the event that you cannot return the survey in-person, please return the survey by mail to:

Rice University – BRC, Hurricane Harvey Registry, MS-460, 6100 Main St, Houston, Texas 77005
